# Effect of thyme extract supplementation on lipid peroxidation, antioxidant capacity, PGC-1α content and endurance exercise performance in rats

**DOI:** 10.1186/s12970-017-0167-x

**Published:** 2017-04-21

**Authors:** Mostafa Khani, Pezhman Motamedi, Mohammad Reza Dehkhoda, Saeed Dabagh Nikukheslat, Pouran Karimi

**Affiliations:** 1 Faculty of Physical Education and Sport Sciences, University of Tabriz, Tabriz, Iran; 2 Faculty of Physical Education and Sport Sciences, Kharazmi University, Tehran, Iran; 3 Neurosciences Research Center, Tabriz University of Medical Sciences, Tabriz, Iran

**Keywords:** Athletic performance, PGC1alpha protein, Reactive oxygen species, Thyme

## Abstract

**Background:**

Athletes have a large extent of oxidant agent production. In the current study, we aimed to determine the influence of thyme extract on the endurance exercise performance, mitochondrial biogenesis, and antioxidant status in rats.

**Methods:**

Twenty male Wistar rats were randomly divided into two groups receiving either normal drinking water (non-supplemented group, *n* = 10) or thyme extract, 400 mg/kg, (supplemented group, *n* = 10). Rats in both groups were subjected to endurance treadmill training (27 m/min, 10% grade, 60 min, and 5 days/week for 8 weeks). Finally, to determine the endurance capacity, time to exhaustion treadmill running at 36 m/min speed was assessed. At the end of the endurance capacity test, serum and soleus muscle samples were collected and their superoxide dismutase (SOD) and glutathione peroxidase (GPx) activity, as well as malondialdehyde (MDA) concentration were measured. Protein expression of PGC-1α, as a marker of mitochondrial biogenesis, was also determined in the soleus muscle tissue by immunoblotting assay.

**Results:**

Findings revealed that the exhaustive running time in the treatment group was significantly (*p* < 0.05) prolonged. Both serum and soleus muscle MDA levels, as an index of lipid peroxidation, had a threefold increase in the thyme extract supplemented group (t_18_ = 8.11, *p* < 0.01; t_18_ = 4.98, *p* < 0.01 respectively). The activities of SOD and GPx of the soleus muscle were significantly (*p* < 0.05) higher in the non-supplemented group, while there was no significant difference in serum SOD, GPx activities, and total antioxidant capacity between groups. Furthermore, thyme supplementation significantly (*p* < 0.05) decreased PGC-1α expression.

**Conclusions:**

Thyme extract supplementation increased endurance exercise tolerance in intact animals, although decrease of oxidative stress and regulation of the PGC-1α protein expression are not considered as underlying molecular mechanisms.

## Background

Reactive oxygen species (ROS) which are produced during aerobic metabolism may oxidize biomolecules and react with different cell sections [1]. Although a physiologically appropriate ROS levels act as a biological messenger, initiating cellular signaling cascades via the process of electron transfer to promote adaptive responses within the body [1], uncontrolled oxidative events can result in lipid, protein and DNA structural damage within cells, leading to an impaired cellular function, a condition generally referred to as oxidative stress [2]. Indeed, imbalances between exercise-induced stress and recovery may impair physiological accommodations, leaving the individual in a maladaptive condition where performance may deteriorate or even decline [3–5]. In fact, extended periods of intensified exercises may result in an accumulation of fatigue, leading to short-term (non-functional over-reaching) and long-term (over-training) decrements in performance capacity [6]. Excessive exercise-induced ROS may also contribute to disruptions in the biological processes, including the metabolic, neuroendocrinological, physiological, psychological, and immunological systems [7].

During exercise, the raised oxidant production in skeletal muscle is regulated by a synergistic response between the endogenous antioxidant system, and the vitamin and mineral antioxidants ingested as part of a well-balanced nutritional diet [8]. The antioxidant system immediately adapts to physical training stimuli to counterbalance the potentially detrimental effects of excessive exercise-induced ROS and to maintain the redox-controlled signaling pathways [9]. However, it has been proposed that those athletes, who sustain an augmented amount of oxidative stress during frequent bouts of exercise, may need a greater extent of exogenous antioxidants to preserve health and enhance performance [10–12]. Therefore, the use of exogenous antioxidant supplements to maintain antioxidant status has been extensively studied. For example, 5 days of supplementation with tart cherry juice (active ingredients: 600 mg phenolic compounds and 560 mg flavonoid compounds) diminished oxidative damage (malondialdehyde and protein carbonyls) and reduced C-reactive protein [CRP] and interleukin [IL]-6, inflammatory cytokines, 48 h post-marathon in 20 well-trained runners [13]. Similar reductions in post-exercise measures of oxidative stress (F-isoprostanes) and muscle damage (creatine kinase, CK) have been discovered in 12 collegiate soccer players who ingested a mixture of antioxidant substances (Resurgex® and Resurgex Plus®) for a 3-week period compared to a placebo (isocaloric equivalent) [14]. These findings defend the hypothesis that antioxidant supplementation may help athletes to cope better with intensified training periods and prevent excessive ROS-induced reductions in performance capacity during exhausting exercise [10, 11].

Natural antioxidant such as the coenzyme Q10, quercetin [15], and resveratrol [16] by having bioenergetics and antioxidant system reinforcement role, induce skeletal muscle and brain mitochondrial biogenesis and thus can delay fatigue and improve exercise performance [17]. The principal reason for such effects seems to be polyphenols compounds. Polyphenols are poly-hydroxylated phytochemical compounds containing two main groups of phenolic acids and flavonoids. Flavonoids are a large family of benzo-[gamma]-pyrone derived low molecular weight phenols, which are present in more fruits and vegetables in different amounts [18–20].

According to available evidence, it seems that flavonoid supplementation increases the performance in endurance activities via an increase in expression of peroxisome proliferator-activated receptor coactivator (PGC-1α) as the “master regulator” of biogenesis [15] and skeletal muscle angiogenesis [21].

Meanwhile, an herbal plant which contains a high flavonoid and antioxidant properties [22], and is widely used in traditional medicine, but its absence in the researches related to sports supplements is felt, is thyme. Thymus migricus Klokov & Desj.-Shost, a member of Lamiaceae family is a Mediterranean native herb which traditionally is used as food additive and its main essence (thymol) is widely used in several medicinal products [22]. Thyme extract has anti-inflammatory, disinfectant, anti-worm, appetizing, sedative [23] and sexually stimulating effects [24]. Regarding the antioxidant properties of thymus migricus Klokov & Desj.-Shost due to its phenolic compounds such as flavonoids and phenolic acids as well as thymol, gamma-terpinene, which are even higher than artificial antioxidants like butylated hydroxytoluene [22], we hypothesized that it may prevent strenuous exercise-induced ROS production and enhance exercise performance, especially in endurance sports. Therefore, the current study aimed to determine the influence of thyme extract supplementation on oxidative stress (lipid peroxidation), antioxidant status, PGC-1α content and endurance performance after a 2-month period of endurance training.

## Methods

### Animals

Twenty male Wistar rats were obtained from Pasteur Institute (Tehran, Iran) at the age of 8 weeks. Experiments were performed after a 3-dayadaptation period. Animals were housed individually in regular cages under 12:12-h light-dark cycle, 22°C temperature, and 50% relative humidity.

Rats were divided randomly into two groups and received standard rat chow containing protein with all the essential amino acids (30%), carbohydrates (40%) and lipids (30%) as well as either water ad libitum (non-supplemented group, *n* = 10) or thyme hydro-alcoholic extract dissolved in distilled water to the desired concentration (400 mg/kg) according to their daily water consumption (30 ml) (supplemented group, *n* = 10). All experimental trials were carried out at the beginning of the dark cycle (19:00 h). Throughout the experiments, there were no significant differences in the mean food consumption (about 20 g per day per rat) and body weight of the animals in the two groups.

### Treadmill performance (maximal endurance capacity)

Rats in both groups were subjected to endurance running on a motorized treadmill 5 days/week. Rats started running for 10 min/day at 10 m/min and 10% grade. The speed and duration were progressively enhanced during the next weeks until each rat was running continuously for a period of 1 h/day at 27 m/min for 8 weeks.

Endurance capacity was assayed by treadmill running to fatigue after the end of the training period. In this step, animals were compelled to run on a motorized treadmill (5/lane) at a speed of 36 m/min and a grade of 8% until they were exhausted [15]. Exhaustion was defined as the inability of the rat to maintain an appropriate pace despite continuous hand prodding for 1 min, at which time the rat was removed from the treadmill, and its run time recorded.

### Hydro-alcoholic extraction preparation

A 30% (W/V) plant material was made in methanol/water (80:20, v/v) at 25 °C, stirred150 rpm for 1 h and filtered through Whatman No. 4 paper. The extra hydro-alcoholic mixture was added to the remnant for extracting the residue. The pooled extracts were dried at 35 °C under reduced pressure (rotary evaporator Büchi R-210, Flawil, Switzerland) and then further lyophilized (FreeZone 4.5, Labconco, Kansas City, MO, USA).

### Sample preparation

Twenty-four hours after the last endurance performance test, rats were deeply anesthetized. Blood samples were withdrawn from the heart and collected into heparinized glass test tubes. Sera and blood cells were separated by centrifugation at 2500 rpm for 10 min at room temperature and subjected to biochemical analyses. The soleus muscles were rapidly excised, frozen, and kept at −80 °C.

### Western blot analysis

The expression of PGC-1α was determined by immunoblotting assay. Briefly, a 10% (W/V) soleus muscle homogenate was made in ice cooled RIPA lysis buffer (Sigma-Aldrich, cod.R0278) containing Protease Inhibitor Cocktail (Roche, cod.11836153001). After mincing by dounce homogenizer and incubating in 4 °C for 30 min, the supernatant was collected by centrifugation at 11,000 × g for 15 min at 4 °C precooled centrifuge (Bekman) and subjected to Bradford assay to determine the protein concentration. The supernatant was mixed (1:1) with Laemmli 2x sample Buffer (50 mM Tris (pH 6.8), 10% Glycerol, 2% SDS, 0.01% Bromophenol Blue) freshly received 2.5% Beta-ME and boiled for 5 min. SDS-PAGE (10% gels) was applied to separate the proteins, afterwards blotted to polyvinylidene difluoride (PVDF) membrane (sigma, cod.427152). Membranes were blocked with PBS-BSA-T buffer, PBST, (8 mmol/L phosphate saline buffer (pH 7.4), 3% BSA, 0.1% Tween 20) at room temperature for 2 h and then exposed to primary antibody against PGC-1α (dilution 1:1000, A cod. ab54481) at 4 °C in a shaker incubator (Behdad, Tehran, Iran) overnight. After undergoing three 5 min of washing steps with PBS-T, the membranes were incubated with horseradish peroxidase (HRP) conjugated goat anti-rabbit antibody (diluted 1:10000) (ab6721, Abcam, UK) for 1 h at 25 °C under gentle agitation. Equal volumes of ECL substrate solution (Bio-Rad, cod. 102030396) and Luminol/enhancer solution (Bio-Rad, cod. 102030394) were used to visualize the protein bands. Image J software was applied to measure the density of the bands.

Local background was subtracted from density volume to obtain a net density of each band. The relative intensity for each sample was calculated by dividing the net intensity of target protein (PGC-1α) to the net intensity of the ß-actin protein band as reference.

### Colorimetric assays

The activities of antioxidant enzymes, including superoxide dismutase (SOD) and glutathione peroxidase (GPx) and concentration of malondialdehyde (MDA) and capacity of total antioxidant of all samples were evaluated by colorimetric assays in the hemolysates, soleus muscle homogenates or sera prepared as briefly follows. After dissection, soleus muscle was weighed and homogenized in 1:3 volume of ice cooled lysis buffer (50 mM Tris-HCl; pH 7.4, 0.5 M sucrose, 0.2 M KCl, 1 mM EDTA) containing cocktail protease inhibitor (Roche, Switzerland) and 0.5 mM DTT. Centrifugation at 12,000 g for 30 min at 4 °C was performed to obtain the supernatant that was used to measure the activity of enzymes.

The total GPx and SOD activities were measured by commercially available Ransel and Ransod kits (Randox company, Crumlin, UK) respectively.

The superoxide dismutase activity was determined based on the rate of inhibition of reaction between2-(4-iodophenyl)-3-(4-nitrophenol)-5-phenyltetrazoliumchloride (I.N.T.) and superoxide radicals (O_2_˙) produced by the xanthine oxidase. Absorbance was read in wavelength of 505 nm. Catalytic activity of GPx in oxidation of GSH (reduced) to GSSH by cumenehydroproxide was measured. GSH was concomitantly produced using glutathione reductase and NADPH. The absorbance was measured by UV filter at 340 nm. The activities of GPx and SOD were expressed as U/g hemoglobin or U/g protein in hemolysate or tissue homogenate respectively.

Thiobarbituric acid (TBA) reactive substances test was applied for measurement of MDA as a major lipid peroxidation product in serum and soleus tissue homogenates. To measure MDA in the soleus tissue, cell lysates were prepared as follows: a 10% (w/v) soleus in 50 mM phosphate buffer (pH 7.4) was made; then, the debris was removed by centrifugation at 12,000 × g for 30 min at 4 °C in a precooled centrifuge (Beckman Coulter, Inc, USA) and stored at −70 °C until analysis. Then thiobarbituric acid (0.6%) and acetic acid (20%) were mixed and incubated in a water bath at 90 °C for 1 h. The mixture was cooled on ice and received a mixture of butanol: pyridine (15:1) plus the samples (diluted 1:40in distilled water) vortexed and centrifuged. Finally, absorbance of the colored supernatant was measured at 535 nm. The results were expressed as nmol/l. Total antioxidant capacity was determined by Randox total antioxidant status kit (cat no. nx 2332, Randox Company, Crumlin, UK) that is performed as followed, RANDOX ABTS® (2,2′-azino-di-[3-ethylbenzthiazoline sulphonate]) was incubated with a peroxidase (metmyoglobin) and H_2_O_2_ to produce the radical cation ABTS®*+. This has a relatively stable blue-green color, which is measured at 600 nm. Antioxidants in the added sample cause suppression of this color production to a degree, which is proportional to their concentration.

Also, serum total cholesterol (TC), triglycerides (TG), high-density lipoprotein cholesterol (HDL-C), and low-density lipoprotein cholesterol (LDL-C) were assayed using commercial available kits (Parsazmoon Inc, Tehran, Iran).

### Statistical analysis

Sample size was determined by PASS sample size software. Resultant data were analyzed using SPSS (version19) through the independent t student test. The results were expressed as mean ± SD. Statistical significance is shown as *p* ≤ 0.05.

## Results

### Effect of thyme supplementation on lipidic indices

Table 1 represents the effect of our treatments on weight and lipid profile of the rats. In the current study, the post weight of the supplemented group did not differ by non-supplemented group (*p* = 0.12) clearly indicated that the thyme extract supplementation did not effect on weight gain or loss. However, the total serum cholesterol and HDL-C are significantly lower in the supplemented group (t_18_ = 3.51, *p* < 0.01; t_18_ = 3.84, *p* < 0.01 respectively).

**Table 1:** Descriptive statistics of performance, weight and some of the lipidic indices

	FEP (Seconds)	Pre-weight (g)	Post-weight (g)	TG (mg/dl)	Total -C (mg/dl)	LDL-C (mg/dl)	HDL-C (mg/dl)
Non-Supp (*n* = 10)	653.00 ± 941.23	250.40 ± 9.32	301.30 ± 20.78	51.90 ± 6.10	68.70 ± 6.07	17.20 ± 4.31	41.13 ± 2.55
Supp (*n* = 10)	3066.00 ± 1631.06*	243.10 ± 8.96	297.00 ± 32.49	48.00 ± 7.80	59.90 ± 5.09*	15.30 ± 2.85	35.00 ± 4.35*

Data are shown as mean ± SD

*FEP* final endurance performance, *TG* triglycerides, *Total-C* total cholesterol, *LDL-C* low density lipoprotein cholesterol, *HDL-C* high density lipoprotein cholesterol, *Non-Supp* non-supplemented, *Supp* supplemented

*p < 0.05 was considered as significant

### Effect of thyme supplementation on maximal endurance capacity

The most obvious finding to emerge from the analysis is that the exhaustive running time of rats in thyme extract supplemented group was significantly prolonged (over four times longer) compared to that of the non-supplemented group (t_18_ = 4.05, *p* < 0.01), Table 1.

### Effect of thyme supplementation on serum and soleus muscle oxidative stress

As shown in Table 2, MDA levels were significantly higher for the treatment group compared to those of the non-treated group both in serum and soleus muscle homogenate (t_18_ = 8.11, *p* < 0.01; t_18_ = 4.98, *p* < 0.01 respectively), which showed elevated lipid peroxidation in the thyme supplemented group.

**Table 2:** The results of independent *t* test within different markers of oxidative stress

	Non-Supp (*n* = 10)	Supp (*n* = 10)	*p*-values
Serum SOD (U/ml)	1262.50 ± 155.95	1160.00 ± 112.33	0.11
Soleus SOD (U/mg)	13.55 ± 1.23	10.46 ± 0.71	0.001^*^
Serum GPx (U/gHb)	68.94 ± 12.14	72.51 ± 14.26	0.55
Soleus GPx (U/mg)	72.48 ± 14.52	56.41 ± 10.02	0.01^*^
Serum MDA (umol/l)	1.10 ± 0.47	3.52 ± 0.82	0.001^*^
Soleus MDA (umol/g)	0.51 ± 0.28	1.79 ± 0.76	0.001^*^
Serum total antioxidant (mmol)	1.34 ± 0.37	1.79 ± 0.63	0.07

Data are shown as mean ± SD

*Non-Supp* non-supplemented, *Supp* supplemented

*p < 0.05 was considered as significant

In addition, the results of experiments showed that there were no significant differences in hemolysate SOD and GPx activity as well as serum total antioxidant capacity between the supplemented and non-supplemented groups (Table 2). However, the activity of both SOD and GPx within the soleus muscle was significantly higher in the non-supplemented group vs. supplemented group. These results are presented in Table 2.

### Effect of thyme supplementation on protein expression of PGC-1α

**Fig. 1:**
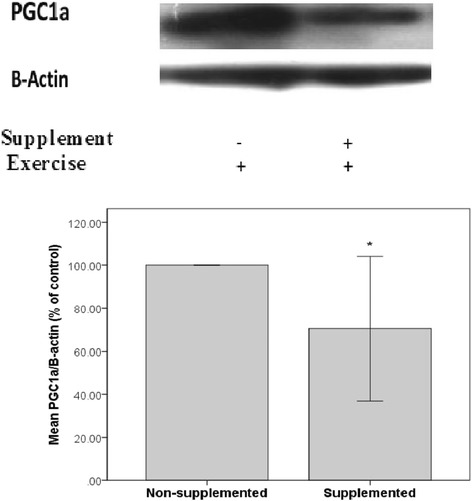
Reduced expression of PGC1a in thyme extract supplemented exercised group in compare with non-supplemented rats. A, Western blot against PGC1a in soleus muscle from Thyme-supplemented and non-supplemented exercised Rats, followed by 24 h rest. B, Quantification of the bands Data has been shown in bottom panel (*n*  =  5/group). *, *p* < 0.05

Thyme supplementation significantly (*p* < 0.05) reduced protein expression of PGC-1α as observed in Fig. 1.

## Discussion

The current research as a pioneer study investigated the possible effects of thymus migricus Klokov & Desj.-Shost extract on endurance performance of rats as well as lipid peroxidation, antioxidant enzymes activity, total antioxidant capacity, muscular content of PGC1-α as a possible mechanism of effect. Results revealed that the exhaustive running time for the treatment group was significantly prolonged showing thyme extract supplementation increased endurance exercise tolerance in intact animals,

Also, the research findings showed that MDA levels in both serum and soleus muscle were significantly higher in the thyme extract supplemented group compared to those of the non-supplemented group, which indicated elevated lipid peroxidation in the thyme supplemented group, the fact that was in contrast with the research hypothesis. Regarding to longer running time (four fold) in the final endurance performance test for supplemented group, it seems that elevated MDA level in the supplemented group was due to the sever exercise (exhaustive running time) rather than the direct effect of thyme supplementation. Study of Yavari et al. 2014 also indicated increased concentration of lipid peroxide after exercise [25]. The elevated levels of mitochondrial ROS during exercise arise from increased tissue and whole-body oxygen consumption. Increased metabolic rate and oxygen consumption by muscle fibers, augments temperature and reduces pH of muscle cells during the exercise, which might also hasten the generation of free radicals [25]. In addition, increased levels of catecholamines during exercise, as well as enhanced release of metmyoglobin from damaged muscles, and reciprocal action of metmyoglobin and methaemoglobin with peroxides throughout exercise have been similarly suggested as mediators of ROS generation [26]. As the intensity of training increases, the ROS production raises leading to endothelial hypoxia, which may cause a decrease in adenosine triphosphate (ATP) sources due to ATP-dependent calcium ionic pump impairment and calcium-dependent proteases activation [27]. The proteolytic activity of these enzymes may result in cleavage of xanthine dehydrogenase and conversion to the oxidase form. Xanthine oxidase catalyzes a reaction which converts xanthine to uric acid and superoxide anions, O_2_˙^-^ [27]. Therefore, ROS production increases and lipid peroxidation may develop. Furthermore, it is possible that the dosage of thyme extract was not enough to prevent lipid peroxidation and MDA production.

The present research also showed that there was no significant difference in serum SOD, GPx, and total antioxidant capacity between the supplemented and non-supplemented groups. However, the activity of both SOD and GPx within the soleus muscle was significantly higher in the non-supplemented group. As mentioned earlier, these result can be explained by the same insight about MDA elevation.

Some studies have reported positive effect of distinct herbal extracts as supplements on antioxidant capacity [28–31], while our results are not in agreement with these studies. This contradiction may be due to the consequence of the tissues specific responses to ROS [32], different duration and intensity of the exercise, dissimilar sample collection methods, using various tissues, or application of different techniques for the measurement of oxidative stress [33].

The soleus muscle is one of the most active muscle groups during running, and it has high mitochondrial content. The augmented production of ROS in the soleus muscle after exhaustive running exercise and the subsequent decrease in the SOD and GPx reducing capacity in the treated group could be explained using body’s antioxidant capacity to quench the damaging radical production [33]. The observed decrease in antioxidant enzyme activity may also reflect allosteric down-regulation of the enzymes, as well as enzyme inactivation assignable to overwhelming oxidative stress.

This study also showed that PGC-1α content of soleus muscle decreased in the supplemented group. A PGC-1α effect on cellular ROS production is mediated by Sirt3, a downstream target gene of PGC-1α [34]. Therefore, lower muscle content of PGC-1α in the supplemented group possibly can explain elevated ROS generation and subsequent higher lipid peroxidation. This finding was consistent with other earlier ones utilizing different kinds of antioxidant supplements, in which PGC-1α, NRF-1, Tfam and cytochrome C were detected to be down-regulated [35, 36]. Regarding down-regulation of PGC-1α in thyme extract supplemented group, it is possible that chronic intake of powerful antioxidant like thyme extract hamper, and even inhibit the improving effect of exercise on physiological adaptations like mitochondrial biogenesis due to decreased levels of ROS in long-term. As mentioned earlier appropriate ROS levels act as a biological messenger, initiating cellular signaling cascades via the process of electron transfer to promote adaptive responses within the body [1]. Indeed, acute exercise induced modest level of oxidative stress can result in PGC-1α up-regulation through the activation of MAP kinases (p38 and ERK1/2), NF-κB, protein kinase B (Akt), mammalian target of rapamycin (mTOR), p70 ribosomal S6 kinase (p70s6K) pathway [37] and thyme supplementation may inhibit these events. This rather contradictory result may be due to the high dosage of long term thyme extract supplementation in the present study which alleviated the positive effects of moderate exercise-induced ROS.

According to the findings of this study, the exhaustive running time of rats in thyme extract supplemented group was significantly prolonged compared to the non-supplemented group, which can be the most practical and predominant finding of this study. This result suggested that thyme supplementation could elevate the exercise tolerance of rats. Although no study has directly examined the effect of thyme extract supplementation on endurance performance, others have reported positive effects of different poly-phenolic compounds [15, 18–20]. Such an improvement in performance seems to be related to cardiovascular, chemo-preventive, immunological, or other adaptations [37] rather than mitochondrial biogenesis (due to lower content of PGC-1α in the supplemented group). In this regard, some researchers also showed that PGC-1α KO mice have regular voluntary running activity in spite of diminished basal mitochondrial respiratory activity [38], indicating that PGC-1α up-regulation may be helpful but not necessary in promoting endurance running performance. This disparity could be related to compensatory accommodations in various tissues because of the down-regulation of the PGC-1α. This result may also be explained by the fact that thyme supplementation has led to decreased hematocrit, hemoglobin and mean corpuscular hemoglobin concentration, all important in determining blood viscosity, (data not reported but available on request). It is well accepted that low viscosity of blood has direct positive correlation with cardiac output, oxygen delivery to the tissue, maximum oxygen consumption, lipid oxidation during exercise, and endurance performance [39]. In this way, thyme extract supplementation can help athletes to improve their endurance capacity. This combination of findings provides some support for the practical effects of thyme supplementation to improve endurance performance regardless of molecular alterations. However, further studies are needed to understand the exact molecular mechanisms. Furthermore, it is suggested to other researchers to take samples at different times (immediately, 24 and 48 h following exercise) and use lower dosage of thyme extract to better understand the effect of this extract on oxidative stress, lipid peroxidation, antioxidants levels and markers of mitochondrial biogenesis.

## Conclusion

Thyme extract supplementation increased endurance exercise tolerance in intact animals, although decreased oxidative stress and regulation of the PGC-1α protein expression are not considered as underlying molecular mechanisms.

## Abbreviations

ATP: Adenosine triphosphate
CAT: Catalase
CK: Creatine kinase
CRP: C-reactive protein
ECL: Enhanced chemiluminescence
EDTA: Ethylenediaminetetraacetic acid
FEP: Final endurance performance
GPx: Glutathione peroxidase
HDL-C: High-density lipoprotein cholesterol
HRP: Horseradish peroxidise
IL-6: Interleukin-6
KO: Knock out
LDL-C: Low-density lipoprotein cholesterol
MDA: Malondialdehyde
Mon: Monocyte
NADP: Nicotinamide adenine dinucleotide phosphate
Non-Supp: Non-supplemented group
PBS-BSA-T buffer: Phosphate buffered saline- Bovine serum albumin-Tween-20 buffer
PGC-1α: Peroxisome proliferator-activated receptor coactivator
PVDF: Polyvinylidene difluoride
RBC: Red blood cell
ROS: Reactive oxygen species
SIRT3: Sirtuin 3
SOD: Superoxide dismutase
Supp: Supplemented group
TBA: Thiobarbituric acid
TG: Triglycerides
Total-C: Total cholesterol
